# Five-year publication rate of clinical presentations at the open and closed American shoulder and elbow surgeons annual meeting from 2005–2010

**DOI:** 10.1186/s40634-016-0059-z

**Published:** 2016-09-09

**Authors:** J. Kay, M. Memon, D. de SA, A. Duong, N. Simunovic, G. S. Athwal, O. R. Ayeni

**Affiliations:** 1 Michael G. DeGroote School of Medicine, McMaster University, Hamilton, Ontario Canada; 2 Division of Orthopaedic Surgery, Department of Surgery, McMaster University, Hamilton, Ontario Canada; 3 Department of Clinical Epidemiology and Biostatistics, McMaster University, Hamilton, Ontario Canada; 4 Roth|McFarlane Hand and Upper Limb Center, St Joseph’s Health Care, Western University, London, Ontario Canada; 5 McMaster University Medical Centre, 1200 Main Street West, 4E15, Hamilton, ON Canada L8N 3Z5

**Keywords:** Publication rate, Clinical presentation, ASES meeting, Evidence-based medicine

## Abstract

**Background:**

The purpose of this study was to evaluate the five-year publication rate of papers presented at both the open and closed American Shoulder and Elbow Surgeons’ (ASES) annual meetings from 2005 to 2010.

**Methods:**

Online abstracts of the presentations at the open and closed ASES annual meetings were independently screened for clinical studies and graded for quality using level of evidence. The databases PubMed (MEDLINE), Ovid (MEDLINE), and EMBASE were comprehensively searched for full-text publications corresponding to these presentations and any paper published within five years of the presentation date was counted.

**Results:**

Overall, 131/266 papers corresponding to the meeting presentations were identified for a five-year publication rate of 49.2 %. Sixty two (48 %) of the papers were published in *The Journal of Shoulder and Elbow Surgeons*, 23 (18 %) were published in *The American Journal of Sports Medicine*, and 20 (16 %) were published in *The Journal of Bone and Joint Surgery*. The mean patient sample size included in presentations with a subsequent full-text publication was higher (154; standard error =27) than the presentations not published (93; standard error = 13) (*p* = 0.039). There was no correlation (*p* = 0.248) between the publication rate and the level of evidence of the presentations.

**Conclusions:**

The publication rate of presentations at ASES meetings from 2005 to 2010 is similar to that reported from other orthopaedic meetings. Studies with large sample sizes should continue to be encouraged, and high quality presentations must consistently be followed up with full-text manuscript preparation in order to maximize the future clinical impact.

## Background

Scientific meetings are important venues that allow for rapid presentation of the latest research advancements to attending audience members. In particular, the American Shoulder and Elbow Surgeons (ASES) association is a leading subspecialty association comprised of shoulder and elbow surgeons and focuses on promoting the highest quality of care available. While many important studies are presented at these meetings, often the ultimate goal of any research project is to publish their report in a peer-reviewed journal. It is critical that all high quality research is disseminated to large audiences by scientific journals to ensure they are factored into important clinical decisions and health policy. If presentations at scientific meetings are not ultimately published in peer-reviewed journals, the issue pertaining to relevance of the research presented at meetings to clinical practice is magnified.

The ASES holds two meetings each year (closed and open for members and non-members, respectively). All submitted abstracts are screened for quality by the program committee before they are accepted for inclusion at the meeting. However, the committee is limited by the minimal information that is provided by the 300-word abstracts that often cannot fully elucidate the quality of the evidence presented. The process of reviewing a study for a meeting presentation is not as rigorous as the peer-reviewing process that is performed by scientific journals. Thus, there are many studies presented at scientific meetings that may never be published in peer-reviewed journals (Bhandari et al. 2002).

It is important to discern how often the presentations at scientific meetings are then published and the factors that contribute to presentations that are not ultimately published. One factor that should be taken into account is the difference in types of papers presented at the closed versus open ASES meetings. Papers at the closed meeting are often more cutting edge and conceptual in nature, and thus these studies are less likely to have immediate clinical application. The closed meeting is designed to allow this type of new research to be presented in a safe setting where experts can provide constructive feedback prior to widespread implementation of new treatments or techniques. At the open meeting, more mainstream topics are selected, and therefore we may expect these papers to have a larger bearing in immediate clinical practice. The rate of publication following presentation at a scientific meeting has been suggested as a measure of the quality of evidence that is presented at the meeting (Daluiski et al. 1998; Kinsella et al. 2015a). It has not been fully elucidated if in fact presentations of higher quality are more likely to have a subsequent full-text publication.

One method to grade the quality of a report is to evaluate the level of evidence of the presentation. The American Academy of Orthopedic Surgeons (AAOS) has standardized this approach for research in orthopedic surgery by creating an evaluation system adopted from the system used by *The Journal of Bone and Joint Surgery* (Wright 2005). This system assigns a particular level of evidence (from I to IV) based on study design with prospective prognostic studies and randomized controlled trials (RCTs) presenting level I evidence (high quality) and case series or reports deemed level IV evidence (low quality). The idea of this classification system is that a more rigorous study design would present evidence that is more reliable in terms of its clinical applications and in its ability to change health policy.

The purpose of this study was to determine the proportion of presentations at the 2005 to 2010 open and closed ASES annual scientific meetings that were ultimately published in a peer-reviewed journal. Furthermore, we evaluated whether various factors such as sample size, level of evidence, meeting type and meeting year had an impact on the publication rate.

## Methods

### Eligibility and analysis of presentations

The methodology used in the present study follows the strategy previously described (Kay et al. 2016). Eligible presentations included clinical paper presentations presented at the 2005–2010 ASES annual open and closed meetings. These years were chosen as they would provide adequate time for publication following their presentation (five years). Five years was used as the evaluation time-frame as several studies have demonstrated that that majority of presentations will be published within five years of the meeting date (Bhandari et al. 2002; Hamlet et al. 1997). Clinical research includes trials and observational studies where there is a direct interaction between an investigator and human subjects. Biomechanical studies, cadaveric studies, technique demonstrations and panel discussions were excluded. *The Journal of Shoulder and Elbow Surgery* (JSES) has electronically published and made available the abstracts for papers presented at the open and closed ASES annual meetings. Two reviewers independently screened the abstracts of the available presentations. At the end of the reviewing process any disagreements were discussed by the two reviewers until a consensus was reached. In order to assess the publication status of the included abstracts, the two reviewers performed detailed searches of PubMed (MEDLINE), Ovid (MEDLINE), and EMBASE in Canada between June 15th 2015 and June 26th 2015 using a slightly modified form of the methodology described by Bhandari et al. (Bhandari et al. 2002). The initial search included the first, second and last author of the abstract. If this search produced only one result matching the intended abstract then the information of this published report was recorded. If the search produced multiple results the Boolean operator ‘AND’ was used to combine the search to include key words from the title of the abstract and additional key words were added until no more than one result remained. If the result obtained was dated less than five years after the corresponding meeting date, (including those published before the date of the meeting) it was included. If reports were published, but not yet printed, the electronic publication date was recorded.

The two reviewers independently evaluated the abstracts and assigned a level of evidence (Level I to IV) to each abstract using the AAOS classification scheme (Wright 2005). Any disagreements that could not be resolved through discussion between the two reviewers were resolved with input from the senior author.

### Data extraction and statistical analysis

Relevant study data was abstracted from the included presentations, including the authors, study title, study type, sample size, study location, level of evidence, publication status, journal of publication and time to publication. These data were recorded in Microsoft Excel 2013 (Microsoft, Redmond, WA). Impact factors of the journals found in this study were 2010 values obtained from the WoS database. In order to assess the inter-reviewer agreement, kappa (k) was calculated for the abstract screening stage as well as for the presentation evaluation stage. Agreement was categorized a priori as follows: k of 0.61 or greater was considered substantial agreement; k of 0.21 to 0.60, moderate agreement; and k of 0.20 or less was considered slight agreement. The proportions and frequencies of the levels of evidence were determined for each meeting and year. Means and standard deviations were calculated for the time to publication results. Chi-squared tests were used in order to test for non-random trends in the publication rates and student t-tests were used when comparing the mean values of quantitative data. A *p*-value of 0.05 or less was considered to be significant. However, when all four level of evidences were evaluated independently, this threshold was adjusted to 0.0125 using the conservative Bonferroni correction for multiple tests (Bland & Altman 1995). All statistics were calculated using Minitab ® statistical software version 17 (Minitab Inc., State College, USA).

## Results

Of the 344 available presentations from 2005 to 2010, 266 were included for assessment. The reviewers in this study reached substantial agreement at the abstract screening and level of evidence evaluation stage with k (and 95 % confidence intervals) of 0.98 (0.94, 1.00) and 0.86 (0.83, 0.89), respectively. No data was available for presentations from the open meeting in 2007 or from the closed meeting in 2010.

**Fig. 1:**
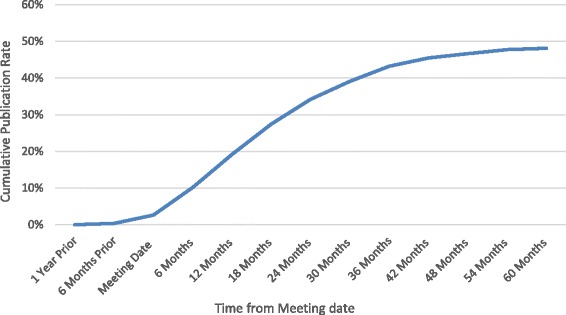
Cumulative graph indicating the full-text publication rate of all presentations at the open and closed ASES annual meetings between 2005 and 2010 at various time points from the meeting date

Overall, 131 of the presentations were ultimately published in a peer-reviewed journal for a 5-year publication rate of 49.2 %. The mean time to publication of the published papers was 18.2 (standard deviation [SD] = 14.6) months (Fig. 1). 7 of the presentations (3 %) were published before the date of the meeting. The 5-year publication rate of presentations at the open meeting was higher (52.8 %) than that of the closed meeting (44.8 %), however, this difference was not significant (*p* = 0.298). Furthermore, the mean time to publication of presentations at the open meeting was shorter (17.0 [SD = 14.0] months) than presentations at the closed meeting (19.4 [SD = 15.1] months), but this difference was also not significant (*p* = 0.362). (Table 1)

**Table 1:** Number of subsequent full text publications separated by meeting type and year of presentation

	2005	2006	2007	2008	2009	2010
Open						
No. of presentations	28	18	N/A	29	29	21
No. published	11	11	N/A	16	16	12
Publication rate	39 %	61 %	N/A	55 %	52 %	57 %
Closed						
No. of presentations	26	29	35	26	22	-
No. published	12	13	15	13	11	-
Publication rate	46 %	45 %	43 %	50 %	50 %	-
Total						
No. of presentations	54	47	35	55	51	21
No. published	23	24	15	29	26	12
Publication rate	43 %	51 %	43 %	53 %	51 %	57 %

**Fig. 2:**
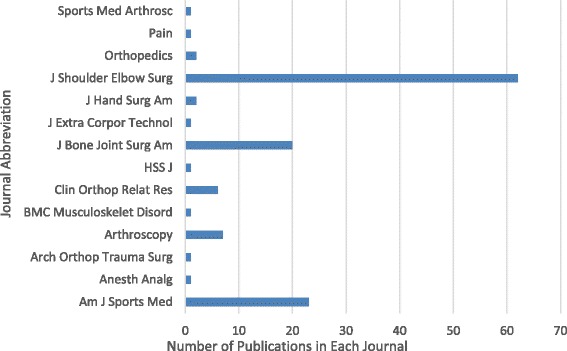
Productivity of journal publications of ASES presentations

In total, 14 peer-reviewed journals published the 129 papers (Table 2). Sixty two (48 %) of the papers were published in *The Journal of Shoulder and Elbow Surgeons* (*JSES*) (2010 Impact factor: 2.311), 23 (18 %) were published in *The American Journal of Sports Medicine* (2010 Impact factor: 3.821), 20 (16 %) were published in *The Journal of Bone and Joint Surgery* (2010 Impact factor: 2.967), and the remainder of the journals published 7 or fewer of the papers (Fig. 2).

**Table 2:** 2010 Impact factors of publishing journals of ASES presentations

Journal	2010 Impact factor
American journal of sports medicine	3.821
Anesthesia & Analgesia	3.274
Archives of orthopaedic and trauma surgery	1.196
Arthroscopy: The journal of arthroscopic and related surgery	3.317
BMC Musculoskeletal disorders	1.941
Clinical orthopaedics and related research	2.116
HSS Journal	0.860
The journal of bone & joint surgery	2.967
Journal of extra-corporeal technology	0.781
Journal of hand surgery	0.868
Journal of shoulder and elbow surgery	2.311
Orthopedics	1.098
Pain	5.355
Sports medicine and arthroscopy review	2.043

Performing a Chi-square analysis revealed that the 5-year publication rate was not significantly associated with the year (*p* = 0.786) or the level of evidence of the presentation (*p* = 0.248). While the publication rate of presentations with level I evidence was the highest (24/42, 57.1 %), the publication rate of level III (27/53, 50.9 %) studies was next followed by level IV studies (64/134, 47.8 %). Presentations designated with level II evidence had a 5-year publication rate of only 37.8 % (14/37). In terms of study type, the publication rate was highest for prognostic studies (54.0 %) followed by therapeutic (47.1 %) and diagnostic (45.5 %) studies. The publication rate was highest for randomized control trials (RCTs) with 59.3 % of presentations being published (Table 3).

**Table 3:** 5-year publication rates and time to publication by level of evidence for the open and closed ASES meetings

	5-Year publication rate	Time to publication Mean (SD) [months]
Open meeting		
Level I	14/22 (63.6 %)	15.2 (15.7)
Level II	6/17 (35.3 %)	10.8 (8.5)
Level III	14/26 (53.8 %)	17.3 (14.4)
Level IV	31/60 (51.7 %)	18.7 (14.7)
Total	66/123 (53.7 %)	17.0 (14.0)
Closed meeting		
Level I	10/20 (50 %)	27.7 (24.6)
Level II	11/23 (47.8 %)	20.8 (20.6)
Level III	13/28 (46.4 %)	17.3 (10.3)
Level IV	33/72 (45.8 %)	17.0 (10.0)
Total	65/143 (45.5 %)	19.4 (15.0)

The mean patient sample size of presentations with a subsequent full text publication was significantly higher than the presentations without a full text publication (*p* = 0.039). The mean sample size of presentations with a corresponding publication was 154 (standard error [SE] =27) while for presentations without corresponding publications the mean sample size was 93 (SE = 13).

## Discussion

Presentations at a scientific meeting can often act as an important resource regarding the available research in a particular field. Not only is the information presented at scientific meetings disseminated to the attending audience, but many orthopedic textbooks cite conference abstracts, allowing the information presented at these meetings the potential to influence clinical decision making (Bhandari et al. 2002). It is therefore important to evaluate the quality of research presented at these meetings. Some consider the rate of subsequent full-text publication as one method that can be used to measure the quality of presentations at scientific meetings (Daluiski et al. 1998; Kinsella et al. 2015b). The 5-year publication rate at the ASES meetings (49.2 %) is comparable to that reported at other scientific meetings. At the AAOS meetings, the publication rate has been reported as 46 %, 44 %, 34 %, and 49 % for the time periods 1990–1992, 1993, 1996 and 2001 respectively (Bhandari et al. 2002; Hamlet et al. 1997; Donegan et al. 2010; Murrey et al. 1999). However, the 5-year publication rate in the present study is lower than that of the shoulder and elbow sessions at the AAOS meeting between 1999 and 2004 which had a reported publication rate of 58 %^5^.

The level of evidence has also been used to assess the quality of presentations at scientific meetings. We assessed whether there was a correlation between the level of evidence and the publication rate of ASES presentations. While studies with level I evidence, particularly RCTs, had the highest 5-year publication rate, there were no significant correlations between level of evidence and the rate of publication at the open and closed ASES meetings. Of note, studies with a level of evidence of II had the lowest publication rate. This finding is consistent with that reported at Arthroscopy Association of North America (AANA) meetings from the same time period, where presentations with level II evidence were found to have the lowest publication rate as well (Kay et al. 2016). A possible explanation for the low publication rate in level II studies may arise from the fact that many level II studies are RCTs that deemed to have a major methodological flaw. The methodological flaws responsible for demoting the level of evidence from I to II may have also prevented publication in a peer-reviewed journal resulting in the relatively low publication rate. More than half of all level I and II presentations have not yet been published in a scientific journal. These findings indicate that the methodological quality of presentations at ASES meetings may not fully predict future publication status. Many presentations of high methodological quality at ASES meetings will not ultimately be published. This is a particularly noteworthy finding, as de SA and colleagues have determined that research of high methodological quality is more likely to be implemented by surgeons in clinical practice (de SA et al. 2015).

Another feature of a study that has been shown to predict future implementation in clinical practice is a large sample size, particularly reports with a sample size greater than 100 (de SA et al. 2015). Our study indicates that presentations with a corresponding full-text publication did have, on average, a significantly larger sample size than the presentations without a corresponding publication. Aside from the quality of a study, the sample size could also indicate the commitment that the authors have devoted towards a study and the likelihood that these authors will dedicate the requisite time needed to prepare a manuscript for peer-reviewed publication. Oftentimes, the authors may intend to publish their manuscript at a later date to allow for reporting on a larger cohort, and instead present preliminary data with smaller sample sizes at scientific meetings. With increased data, the results of the study might change providing different barriers to full-text manuscript preparation. Furthermore, because time is spent increasing the sample size, the investigators may change over the study’s course which provides an additional challenge for co-authors to prepare a manuscript. While sample size is likely considered as one of many factors when selecting abstracts for conference presentations, our results would indicate that additional weight towards the sample size during the grading process may correspond to a higher publication rate. This can help to ensure that information presented at the meeting will correspond to that which will ultimately be used as the basis for clinical and surgical management.

Authors might be influenced by the perceived quality of the journal when deciding where to submit a manuscript. The quality of journals is typically assessed using impact factors. The impact factor of a journal in any given year is determined by finding the mean number of citations received in a given year for all articles published in the two preceding years (Saha et al. 2003). While there are well known limitations involving the use a journal’s impact factor as a precise measure of its quality, impact factors are valued quite strongly with regards to the perceived quality of the journal (Amin & Mabe 2003). The top four publishing journals in the present study (in terms of number of publications) are all among the journals with the highest 2010 impact factors. A preference to publish in journals with higher impact factors may be contributing to these results.

One factor that greatly affects the quality of presentations at conferences is the abstract review committee. The peer-review process is fairly subjective, and it is likely that there are factors other than methodological quality of a study that affect whether or not a meeting presentation will be accepted, and thus followed by a full-text publication in a peer-reviewed journal (Relman 1990). In fact, of presentations at the AAOS meeting that had not yet been published, only 25.2 % were actually submitted and rejected from a peer-reviewed journal according to Sprague and colleagues. This follows from the finding that less than two-thirds of presentations at the AAOS meeting were ultimately submitted to a peer-reviewed journal (Sprague et al. 2003). Of the presentations that were not submitted to a peer-reviewed journal, the three most common reasons given for the lack of submission was insufficient time to prepare a manuscript, the manuscript was still in progress and co-authors moving or changing institutions (Sprague et al. 2003). A similarly low submission rate of presentations from ASES meetings might contribute to the lack of correlation between the level of evidence and publication rate in the present study. It is vital that authors prepare manuscripts for all presentations to ensure that journals can truly select the highest quality research for dissemination.

This study is the first to assess the publication rate of the presentations presented at the ASES annual scientific meetings. Multiple years of data were included and the method of data extraction was thorough and systematic. However, this study is limited by the possibility that published full-text articles may not have been identified by the PubMed (MEDLINE), Ovid (MEDLINE), and EMBASE search (such as journals that are not indexed by the these databases), resulting in an underreporting of the true publication rate, although a comprehensive search methodology was used. Nevertheless, the publication rate reported in this study is similar to the publication rate reported from other orthopedic meetings (Bhandari et al. 2002; Daluiski et al. 1998; Hamlet et al. 1997; Donegan et al. 2010; Murrey et al. 1999). It is common for presentations to be submitted to, and presented at multiple meetings. Bhandari et al. found that roughly 1 in 5 presentations at the 2001 Canadian Orthopaedic Association were also presented at the 2001 or 2002 AAOS annual meetings (Bhandari et al. 2005). The present study observed only the presentations submitted to the ASES meetings. Future research should evaluate whether studies presented at multiple meetings would have a different publication rate.

## Conclusions

The five-year publication rate of research presented at ASES meetings between 2005 and 2010 is similar to the publication rate detected from other orthopaedic meetings, as reported in the literature. Importantly, the publication rate was not correlated with the methodological quality indicating that a significant portion of the highest quality evidence is not being disseminated to clinicians and influencing health policy. Studies with a large sample size should continue to be encouraged, and high quality presentations must consistently be followed up with full-text manuscript preparation in order to maximize the future clinical impact.

## Abbreviations

ASES: American shoulder and elbow surgeons’
AAOS: American Academy of Orthopedic Surgeons
RCTs: Randomized controlled trials
JSES: The journal of shoulder and elbow surgery
SE: Standard error
